# A Stirring Appeal to the Profession in Behalf of the Order, by Dr. Day. President Louisiana P. M. B. A.

**Published:** 1888-03

**Authors:** 


### P. M. B. A. OF TEXAS.

It is proposed to hold a meeting of the members of this order in Galveston during the time of the convention of the State Medical association; and it is hoped that an interest may be awakened amongst the profession which shall swell the ranks and increase the usefulness of the organization.

In anticipation of that meeting, and in the direction of awakening increased interest in the matter, it is opportune here to say something on the subject; but having read with great interest the following appeal to the physicians of Louisiana in a similar cause by their venerable and worthy president, Dr. R. H. Day, we conclude that we can do no better than to reproduce it. It goes to the center, and if human language can stir the average medical heart, this touching appeal should meet with a hearty rosponse, addressed here to the physicians of Texas. Only change the names—substitute those of some of our recently bereaved ones, and take the matter home to yourselves—gentlemen of medical profession of the great state of Texas.

*To the Physicians of Louisina and of Texas.*

The demise of Dr. David McCaa, late a member of the Physicians Mutual Benevolent Association of Louisiana, leaving behind him four small motherless children, entirely helpless and unprovided for, impels me once more to appeal to your higher and refined natures in behalf of the widows and orphans of your brother physicians.

The appended receipt (a true copy of the original on file) will show that our physicians have only provided the small sum of ninty-nine dollars to give to the little helpless children of Dr. David McCaa, deceased—a man of merit and worth in his profession, a native of Louisana and a graduate of the State Medical University in New Orleans. I confess I feel humiliated at this demonstation of the lack of sympathy and brotherly kindness among the physicians of our State one for the other—if not this, a want of interest in, or culpable indifference to, the comfort and well-being of each other's families.

Had our 800 or 1000 regular physicians in Louisana been true to each other, and joined our Mutual Benevolent Association promptly when first organized, as was their obvious duty, instead of giving to Dr. McCaa's bereaved and helpless children $99, we would have been enabled to contribute $2400 or $3000; and that too, with the insignificant tax of only $4 to each of us, a sum which, I venture to assert, is more than ten times *uselessly spent* every year by ninety-nine hundreths of our physicians.

Brother physicians, look at the manifest truth as it is. Picture in your minds and hearts these four little bereaved orphans of a worthy decased brother, unprovided for and thrown in tender infancy upon the care of strangers, and reflect how much good you could have done,and did not do it! How many little hearts you could have made happy, and did not do it, how many sweet prayers from tender grateful hearts would have ascended to heaven to call down rich blessings upon you and our noble profession, and you did nothing to elicit the invocation; and how the good people among whom you live would have heralded your noble benevolence and brotherly love, and voiced and echoed your praises, but, alas, you did nothing to deserve it! And your professions of brotherly love one for the other are as "*sounding brass* or a *tinkling cymbal.*"

Brother physicians, let your hearts be moved by the noble instincts of christianized humanity, and by the fraternal principles of our noble profession. and delay no longer—join our band of true brothers at once—and let us go up to our meeting in Monroe next April with hundreds of new members added to our Benevolent Association. Don't be afraid that some old man in the profession will die before you do, and his family get the benefit first, but come in now, live as long as you can and do all the good that lies in your power, and your brothers who are alive will see to it, when you have passed from the earth, that your widow and children shall receive an hundred fold more than all you had contributed during your life time.

Fraternally,

RICHARD H. DAY, M. D.
*President P. M. B. A.*

